# Computational Modeling of the Interactions between DPP IV and Hemorphins

**DOI:** 10.3390/ijms25053059

**Published:** 2024-03-06

**Authors:** Priya Antony, Bincy Baby, Amie Jobe, Ranjit Vijayan

**Affiliations:** 1 Department of Biology, College of Science, United Arab Emirates University, Al Ain P.O. Box 15551, United Arab Emirates; 2 The Big Data Analytics Center, United Arab Emirates University, Al Ain P.O. Box 15551, United Arab Emirates; 3 Zayed Center for Health Sciences, United Arab Emirates University, Al Ain P.O. Box 15551, United Arab Emirates

**Keywords:** dipeptidyl peptidase IV, hemorphins, diabetes, molecular dynamics

## Abstract

Type 2 diabetes is a chronic metabolic disorder characterized by high blood glucose levels due to either insufficient insulin production or ineffective utilization of insulin by the body. The enzyme dipeptidyl peptidase IV (DPP IV) plays a crucial role in degrading incretins that stimulate insulin secretion. Therefore, the inhibition of DPP IV is an established approach for the treatment of diabetes. Hemorphins are a class of short endogenous bioactive peptides produced by the enzymatic degradation of hemoglobin chains. Numerous in vitro and in vivo physiological effects of hemorphins, including DPP IV inhibiting activity, have been documented in different systems and tissues. However, the underlying molecular binding behavior of these peptides with DPP IV remains unknown. Here, computational approaches such as protein–peptide molecular docking and extensive molecular dynamics (MD) simulations were employed to identify the binding pose and stability of peptides in the active site of DPP IV. Findings indicate that hemorphins lacking the hydrophobic residues LVV and VV at the N terminal region strongly bind to the conserved residues in the active site of DPP IV. Furthermore, interactions with these critical residues were sustained throughout the duration of multiple 500 ns MD simulations. Notably, hemorphin 7 showed higher binding affinity and sustained interactions by binding to S1 and S2 pockets of DPP IV.

## 1. Introduction

Dipeptidyl peptidase IV (DPP IV), also known as cluster of differentiation 26 (CD26), is a multifunctional transmembrane serine protease that is widely expressed in a variety of mammalian tissues, including endothelial cells, epithelial cells and lymphocytes [1]. DPP IV modulates the activity of several peptide hormones, chemokines and neuropeptides by cleaving Xaa-Pro or Xaa-Ala from their N terminus. Specific cleavage of polypeptides by DPP IV results in the regulation of multiple hormones and peptides, including glucagon-like peptides (GLP1) and glucose-dependent insulinotropic peptides (GIP), two important insulin-secreting incretins. GLP1 and GIP signaling plays a potent role in glucose homeostasis by enhancing glucose-induced insulin secretion and suppressing glucagon secretion. DPP IV is important in maintaining the tight control of glucose levels by terminating the secretion of these incretins. As the role of DPP IV in clearing these intestinal peptides has been firmly established, DPP IV inhibitors are widely used for the treatment of type 2 diabetes mellitus(T2DM) [2].

**Figure 1:**
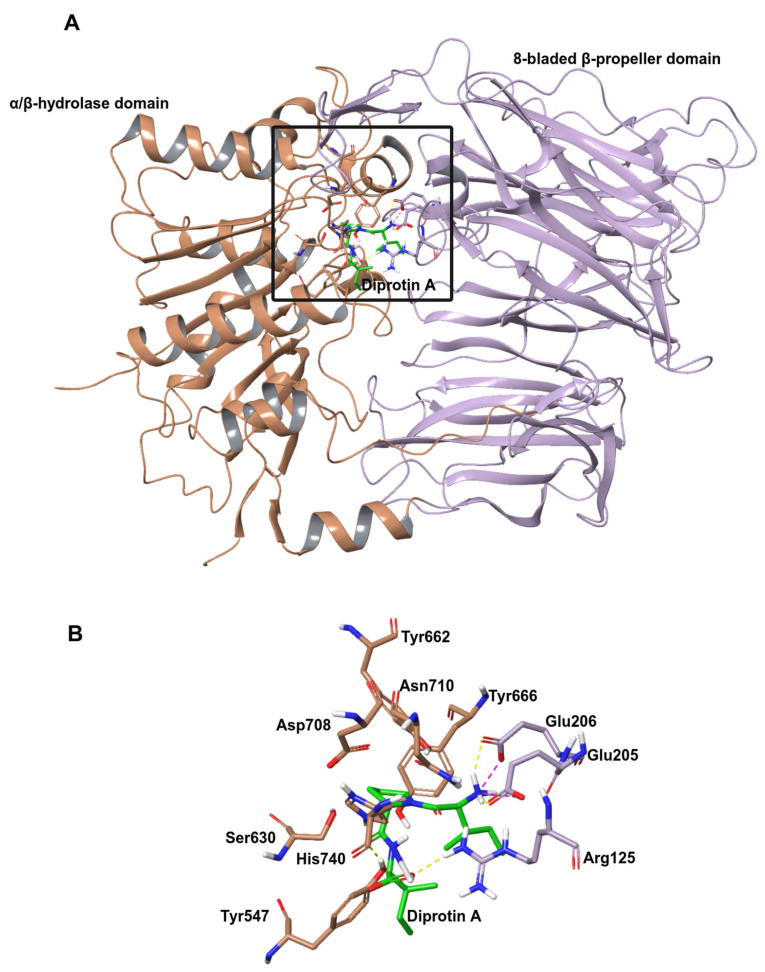
Structure of human DPP IV (PDB ID:1WCY) [6]. (**A**) Structure of DPP IV monomer with bond ligand diprotin A. The α/β hydrolase domain is brown, the β-propeller domain is lilac, and the ligand is green. The interface between these two domains forms a central cavity within the black box. (**B**) Active site residues of the protein.

As several X-ray crystallographic structures of the DPP IV with small molecule inhibitor complex have been published (for e.g., Protein Data Bank (PDB) structures with ID 1WCY, 6B1E, 5I7U and 4N8D), the molecular level interaction of the protein with inhibitors has been well studied [3,4,5,6]. DPP IV is a single polypeptide chain consisting of 766 amino acids that contain an N-terminal β-propeller domain and a C-terminal α/β hydrolase domain (Figure 1). The interface between these two domains forms a central cavity that encapsulates the active site of the protein. The protein harbors a relatively large substrate binding pocket composed of four subsites (S1, S1′, S2 and S2 extensive), and most existing inhibitors stably bind to the protein by making interactions with multiple subsites. In particular, central scaffolds of oral antidiabetic drugs gliptins mainly interact with the S1 and S2 pockets to inhibit the activity of the enzyme. Moreover, additional interactions at the S2 extensive subsite contribute to improved affinity [7].

While several studies have looked at DPP IV-small molecule inhibitor interactions, very little is known about how peptides access and bind to the active site of the enzyme [6,8,9,10] (Figure S1). Small molecule inhibitors interact inside the hydrophobic cavity of DPP IV made up of Arg125, Glu205, Glu206, Phe357, Tyr547, Ser630, Tyr662, and Tyr666. [11,12]. Structural studies suggest that the binding site of DPP IV is accessible in two ways—either via the opening of the propeller domain or through an opening in the side formed at the interface of the β-propeller domain and α/β hydrolase domain [13]. Several features suggest that most peptides access the active site via the large side opening as it is the shortest and most accessible way to reach the region. Even though the side opening of the DPP IV is large, the active site is located in a small region within the cavity so only elongated peptides, unfolded or partly unfolded, can reach the site [14]. It was also reported that the negatively charged cleft, with a conserved double Glu motif (Glu 205, Glu 206) located on the short helix insertion of the beta-propeller domain, could attract the positively charged N terminus of the peptide substrate [13]. This orients the peptide in the right way for cleavage. However, binding orientation and pose could vary based on the amino acid sequence of the peptide. For instance, if the proline is located in the third position from the N-terminal region of the peptide, the binding pattern differs from when it is in the penultimate position. This suggests that the spatial arrangement and interactions of the peptide with the binding site are influenced by the specific positioning of the proline within the sequence [8,9].

Peptide therapeutics represent a well-known class of promising treatments, with around 80 marketed drugs and an additional 150 peptide-based drug candidates in clinical trials [15]. They are bioavailable and stable molecules capable of exploring large contact surface areas of the protein [16,17]. Hemorphins are a group of endogenous bioactive peptides produced by the physiological or pathophysiological degradation of all hemoglobin chains except the alpha chain [18,19]. These peptides are short, ranging from 4–10 amino acids in length and share a central tetrapeptide core (Tyr-Pro-Trp-Thr) [20]. Numerous in vitro and in vivo physiological effects of hemorphins have been documented in different systems and tissues. These molecules work as opioid receptor ligands by exhibiting affinity for μ-, δ- and k-receptors and show antinociceptive activity [21]. Effects of hemorphins on the renin-angiotensin system (RAS), the kinin-kallikrein system (KKS) and insulin-regulating aminopeptidase (IRAP) are also documented [22,23]. Apart from this, these peptides are also reported to modulate the activity of calcineurin and Ca^2+^/calmodulin-dependent systems [24]. 

Multiple DPP IV inhibiting bioactive peptides have been identified from various sources by different research groups [25]. Based on these published data, the current consensus is that peptides having proline or alanine as the second amino acid from the N terminus exhibit high inhibitory activity. As hemorphins are natural bioactive peptides bearing the Tyr-Pro motif at the N-terminus, it is very probable that hemorphins can interact with DPP IV [26]. However, the precise molecular binding behavior of hemorphins has not been elucidated yet. Currently, computational techniques are invaluable in predicting, designing, modifying and optimizing putative peptides [26,27,28]. In this study, flexible protein–peptide molecular docking was employed to provide insights into the binding orientation of hemorphins in the active site of DPP IV. The lead peptides exhibiting promising interactions with the protein were further analyzed using extensive molecular dynamics (MD) simulations to study their stability and dynamics at the molecular level. The findings of this study indicate that hemorphins lacking the hydrophobic residues LVV and VV at the N terminal region strongly bind to the conserved residues in the active site of DPP IV. Furthermore, interactions with these critical residues were sustained throughout the duration of multiple 500 ns MD simulations. Future research on hemorphins using in vitro and in vivo analysis will shed additional insight into their underlying inhibitory properties. These efforts will contribute to the development of novel therapeutic peptides for the management of type 2 diabetes.

## 2. Results

### 2.1. Molecular Docking

Eighteen hemorphins, including camel hemorphin sequences that differ from other hemorphins at one position, were docked flexibly to the catalytic site of the DPP IV protein. Table 1 provides the binding score and amino acid residues of DPP IV that interacted with the hemorphins. Based on the docking score (GlideScore or GScore), binding free energy and peptide binding orientation, it was observed that hemorphins lacking N terminal LVV and VV residues exhibited better scores and similar binding orientation in the active site of the protein. On the other hand, LVV and VV hemorphins were positioned differently in the DPP IV catalytic pocket compared to the other hemorphins (Table S2 and Figures S4 and S5). Therefore, peptides with better binding scores, MM-GBSA binding energy and similar binding orientations were further analyzed.

#### 2.1.1. Docking of Hemorphins to DPP IV

The binding affinities and poses of hemorphins (H7, H6, H5 and H4) with DPP-IV were evaluated, revealing different GScore and MM-GBSA binding energy values for each ligand. H7 (YPWTQRF) had a GScore of −11.44 kcal/mol and an MM-GBSA binding energy of −119.64 kcal/mol in the identified binding pose, whereas the best-docked pose of H6 (YPWTQR) in DPP IV had a GScore of −10.53 kcal/mol and an MM-GBSA binding energy of −92.92 kcal/mol. The best binding conformation of H5 (YPWTQ) had a GScore of −10.76 kcal/mol and an MM-GBSA binding energy of −99.12 kcal/mol and the GScore and MM-GBSA binding energy obtained for the best binding pose of H4 (YPWT) was −7.61 kcal/mol and −84.98 kcal/mol, respectively.

**Figure 2:**
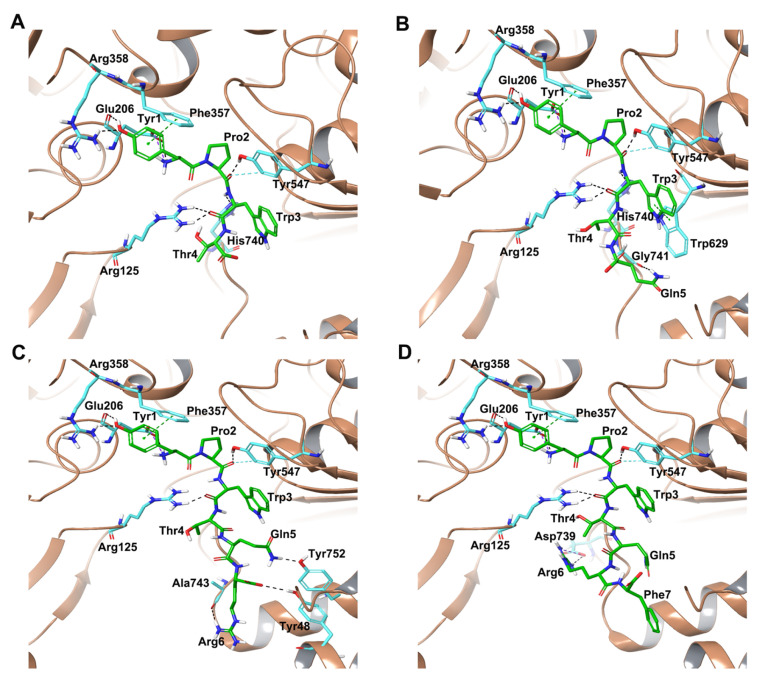
Interactions between DPP IV and hemorphins obtained from molecular docking. (**A**) DPP IV-H7, (**B**) DPP IV-H6, (**C**) DPP IV-H5, (**D**) DPP IV-H4.

Hemorphins H4, H5, H6 and H7 bound in a similar pose interacting with critical residues in the active site of DPP IV. The tetra peptide core (YPWT) of the hemorphins exhibited a similar pattern of interactions with DPP IV. The N terminal Tyr1 of all hemorphins formed salt bridge and hydrogen bond interactions with Glu206 and Arg358 residues of the protein. Additionally, Tyr1 was also observed to form a π-π interaction with Phe357. Furthermore, Pro2 and Trp3 of these hemorphins established hydrogen bonds with Tyr547 and Arg125, respectively. In H5, Pro2 was also found to interact with His740 and Trp3 formed π–π interaction with Trp629. Additionally, in H4, Pro2 and Trp3 formed hydrogen bonds with Arg125, Tyr547 and His740, which are important S2 pocket residues (Figure 2D and Figure S2A).

Apart from these interactions, the guanidino group of Arg6 in H7 established a hydrogen bond and salt bridge with Asp739 (Figure 2A and Figure S2D). In H6, the Glu5 interacted with Tyr752 of the protein by forming hydrogen bond interactions. Additionally, the C terminal Arg6 interacted with Tyr48, His748 and Ala743 by forming hydrogen bonds (Figure 2B and Figure S2C). In H5, Glu5 established a hydrogen bond with Gly741 (Figure 2C and Figure S2B). The hemorphins ability to stay in the active site was further aided by several hydrophobic interactions with important residues.

#### 2.1.2. Docking of Camel Hemorphins to DPP IV

The binding affinities and poses of camel hemorphins with DPP IV were also evaluated. The best pose of camel H7 in DPP IV had a GScore of −10.54 kcal/mol and an MM-GBSA binding energy of −113.28 kcal/mol, and camel H6 was observed to have a GScore of −10.07 kcal/mol and an MM-GBSA binding energy of −85.99 kcal/mol. The binding of camel H5 with DPP IV produced a Gscore of −9.42 kcal/mol and an MM-GBSA binding energy of −102.88 kcal/mol.

**Figure 3:**
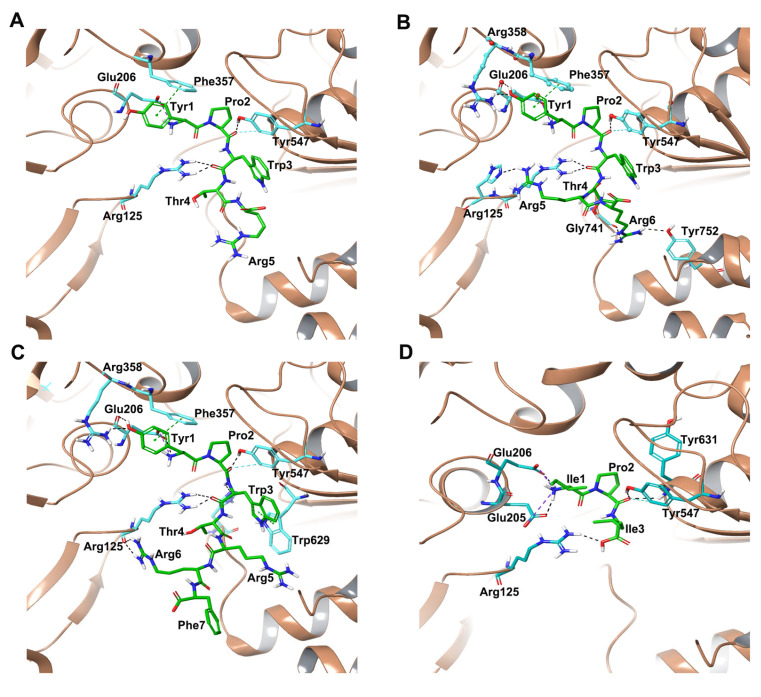
Interactions between DPP IV and camel hemorphins obtained from molecular docking. (**A**) DPP IV-Camel H7, (**B**) DPP IV-Camel H6, (**C**) DPP IV-Camel H5, (**D**) DPP IV-Diprotin A.

Similar to non-camel hemorphins, the tetrapeptide core (YPWT) exhibited similar interactions in the active site of the protein. In camel H7 and camel H6, the backbone of Tyr1 at the N terminus was observed to form a salt bridge and hydrogen bond with Glu206, while in camel H5, N terminal Tyr1 residue produced only a salt bridge with Glu206 (Figure 3C and Figure S3A). Aside from this interaction, the phenolic sidechain of Tyr1 in camel hemorphins formed π-π interaction with Phe357. In camel H7, the hydroxyl group of Tyr1′s sidechain formed hydrogen bonds with Arg358 and Glu206 residues. The Pro2 of the camel hemorphins was found to establish a hydrogen bond with Tyr547 residue, an essential amino acid regulating the catalytic mechanism of the protein. In camel H7, Pro2 formed an additional hydrogen bond with His740. Trp3 of the camel hemorphin peptides established hydrogen bond with Arg125 (Figure 3C and Figure S3C). In camel H7, Trp3 formed an additional π-π interaction with Trp629 of the protein.

Apart from these contacts, the guanidino group of Arg6 in camel H7 established two hydrogen bonds with the backbone carboxyl group of Arg125 of the protein (Figure 3A and Figure S3C). In camel H6, the guanidium group of Arg5 interacted with the imidazole ring of His126 by making hydrogen bonds whereas the guanidino group of Arg6 was found to interact with Gly741 and Tyr752 (Figure 3B and Figure S3B). Additionally, hydrophobic, polar and electrostatic interactions between the peptide and critical DPP IV residues facilitate the binding of the peptide in the active site.

As a positive control, molecular docking analysis was performed for the DPP IV- diprotin A complex (PDB ID:1WCY). Diprotin A (Ile-Pro-Ile, IC_50_ = 4.21 ± 2.01 μM) is the most potent competitive inhibitor of DPP IV that binds to the substrate-binding site of DPP IV [29,30]. Docking reproduced the experimental pose of diprotin A with an RMSD value of 0.190 Å (Figure S6). Diprotin A produced a docking score of −8.05 kcal/mol and MM-GBSA binding energy of −83 kcal/mol. The N terminal of Ile1 formed a hydrogen bond and salt bridge with Glu205 and Glu206, while Pro2 interacted with Tyr547 and Tyr631 by forming hydrogen bonds. Ile3 interacted with Arg125 by forming a hydrogen bond (Figure 3D and Figure S3D).

In all shortlisted camel and non-camel hemorphin peptides, core residues were observed to interact with amino acids in the catalytic site of DPP IV by forming an extensive network of hydrogen bonds, as well as hydrophobic and electrostatic interactions, as shown in the 2D ligand interaction diagrams (Figure 2, Figure 3, Figure S2 and Figure S3).

### 2.2. Molecular Dynamics Simulations 

To evaluate the stability of the docked poses, multiple 500 ns MD simulations of the protein–peptide complexes were performed using Desmond.

#### 2.2.1. MD Simulations of DPP IV-Hemorphin Complexes

**Figure 4:**
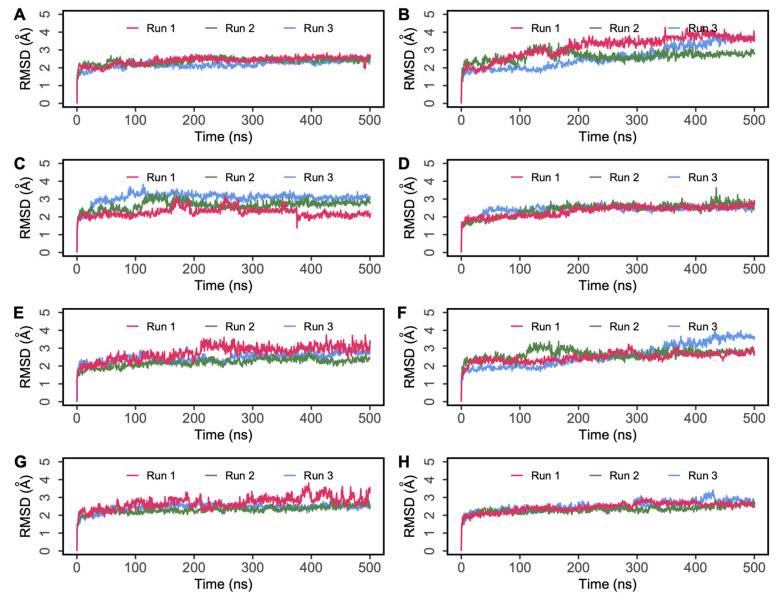
Root mean square deviation (RMSD) of protein Cα atoms obtained from 500 ns simulations of (**A**) DPP IV-H7, (**B**) DPP IV-Camel H7, (**C**) DPP IV-H6, (**D**) DPP IV-Camel H6, (**E**) DPP IV-H5, (**F**) DPP IV-Camel H5, (**G**) DPP IV-H4, (**H**) DPP IV-Diprotin A.

The root mean square deviation (RMSD) of protein C-alpha (Cα) atoms was evaluated to study the difference in protein conformation in each frame of the MD trajectory when compared to the initial structure. An assessment of the RMSD provides insights into the enzyme’s flexibility and conformational changes induced upon ligand binding. Figure 4 shows the change in protein RMSD in the presence of hemorphin peptides in 500 ns MD simulations. For all peptide complexes (DPP IV-H7, DPP IV-H6, DPP IV-H5 and DPP IV-H4), some degree of fluctuation was observed during the initial stage of the simulation, after which it plateaus to a stable level. In the DPP IV-H7 complex (Figure 4A), the system was found to be stable in all three simulation runs. This was expected as based on the GScore and MM-GBSA values, H7 had a higher binding affinity and produced sustained interactions with DPP IV. In the DPP IV-H6 complex (Figure 4C), slight fluctuations were observed from the start to 200 ns, and the system stabilized thereafter. In the DPP IV-H5 complex (Figure 4E), even though a slight variation was observed around 200 ns in run 1, the system subsequently stabilized. Finally, in the DPP IV-H4 complex (Figure 4G), the system was found to be stable around 2 Å in runs 2 and 3, whereas run 1 exhibited some amount of fluctuation in RMSD around the mean value. The variations of RMSD values in three runs are expected as the inherent stochastic nature of the simulations can result in different trajectories and conformational ensembles, contributing to the variability in RMSD values across the runs. Overall, these results suggest that the binding of hemorphins with human DPP IV was stable.

**Figure 5:**
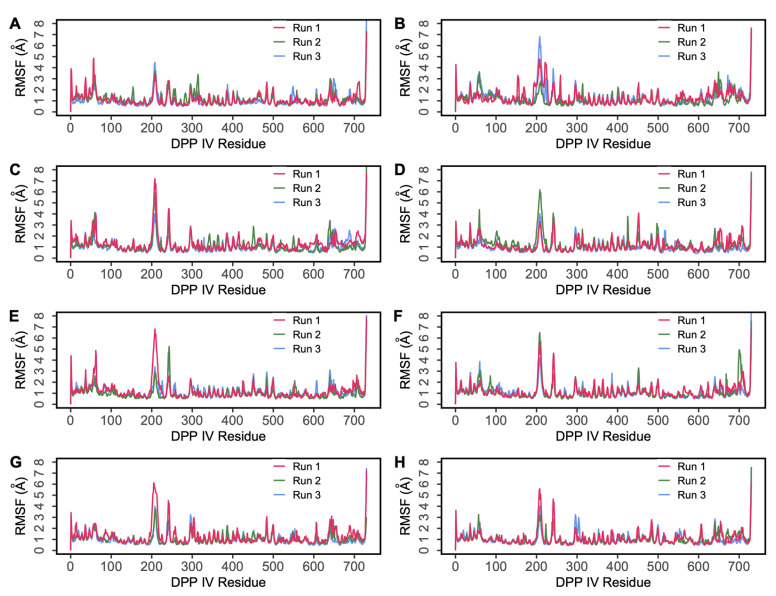
Root mean square fluctuation (RMSF) of protein residues obtained from 500 ns simulations. (**A**) DPP IV-H7, (**B**) DPP IV-Camel H7, (**C**) DPP IV-H6, (**D**) DPP IV-Camel H6, (**E**) DPP IV-H5 (**F**) DPP IV-Camel H5, (**G**) DPP IV-H4, (**H**) DPP IV-Diprotin A.

Root mean square fluctuation (RMSF) reveals the fluctuation of residues during the simulation process around their average position, which can be used to assess the dynamic stability of the system. Figure 5 presents the RMSF values of DPP IV during the 500 ns MD simulations. The RMSF plot shows similar fluctuations for all four systems (Figure 5A,C,E,G). However, there was an increased RMSF observed around 220–250 residues, which are predominantly occupied by loops. Compared to the RMSF graphs of complexes involving H6, H5 and H4, the fluctuations were lower in the H7 complex indicating the ligand was bound stably in the binding pocket of the protein. The ligand RMSF of non-camel hemorphins were also analyzed. A low level of fluctuation was observed for all the peptide ligands indicating stable binding (Figure S7). Here, the monomeric structure of DPP IV was used in all simulations. It was observed that the monomers exhibited fluctuations that resembled those of dimers [31].

**Figure 6:**
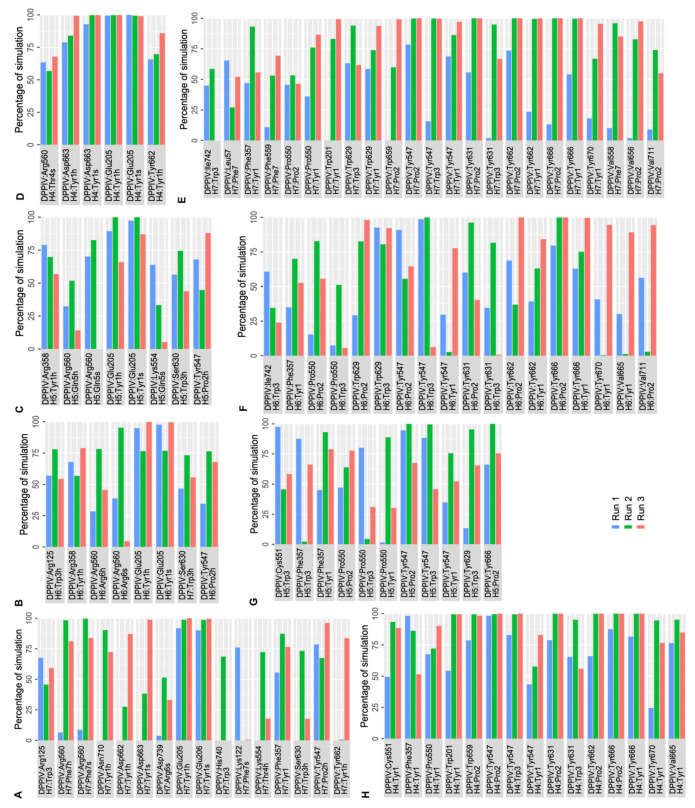
Percentage of simulation time during which intermolecular polar and hydrophobic contacts were retained between DPP IV and non-camel hemorphin peptides in the 500 ns systems. (**A**) Polar interaction of H7 with DPP IV, (**B**) polar interaction of H6 with DPP IV, (**C**) polar interaction of H5 with DPP IV, (**D**) polar interaction of H4 with DPP IV, (**E**) hydrophobic interaction of H7 with DPP IV, (**F**) hydrophobic interaction of H6 with DPP IV, (**G**) hydrophobic interaction of H5 with DPP IV, (**H**) hydrophobic interaction of H4 with DPP IV.

Protein–peptide interaction plots were used to identify the stability of residue-level polar and hydrophobic interactions generated by non-camel hemorphin peptides with DPP IV. As the peptide with the highest GScore and MM-GBSA binding free energy with DPP IV, H7 had the most interactions in the active site of DPP IV, occupying the protein’s S1, S1′, S2 and S2 extensive pockets. The N terminal backbone of Tyr1 maintained hydrogen bond and salt bridge interactions with Glu205 and Glu206 of the S2 pocket throughout the simulations. Additionally, the backbone of this residue was also found to be engaged in hydrogen bond interaction with Tyr662 and Phe357, along with a salt bridge contact with Asp663. This residue was also observed to form consistent hydrophobic interactions with S1, S1′, S2 and S2 extensive pocket residues such as Trp201, Phe357, Tyr547, Pro550, Tyr666, Tyr662, Trp629 and Tyr670. Apart from maintaining hydrogen bonds with Tyr547, Pro2 was found to be engaged in hydrophobic interactions with Pro550, Tyr547, Val656, Tyr666, Tyr631, Trp659 and Val711. Trp3 maintained its polar contact with Arg125, Ser630 and His740 residues in the catalytic triad and hydrophobic interactions with Tyr547 and Trp629 residues. Additionally, Thr4 engaged in a hydrogen bond interaction with Lys554 for almost 75% of the simulation time. The C terminal Phe5 residue makes hydrogen bonds and salt bridge interaction with Arg560, and maintains a consistent hydrophobic interaction with active site residues (Figure 6A,E).

In H6 simulations, the backbone of N terminal Tyr1 followed an interaction pattern similar to H7 by forming hydrogen bonds and salt bridge interaction with Glu205. Additionally, this residue formed stable hydrophobic interactions with Trp201, Phe357, Tyr547, Val665, Tyr666, Tyr662 and Tyr670. Pro2 formed a hydrogen bond with Tyr547 and interacted hydrophobically with crucial residues including Pro550, Trp629, Tyr547, Tyr631, Tyr662, Tyr666, Val665 and Tyr670. Trp3 maintained hydrogen bonds with Arg125, as well as Ser 630. Trp3 maintained hydrophobic contact with Cys551, Tyr547, Tyr631, Ile742, Ala743 and Trp629. The backbone of Arg6 retained a hydrogen bond and salt bridge interaction with Arg560 (Figure 6B,F).

The N terminal residues of H5 mediate the majority of active site interactions with DPP IV. In H5 simulations, the backbone of Tyr1 retained a hydrogen bond and salt bridge interaction with Glu205. Tyr1 also interacted with Arg358 by forming hydrogen bonds. This residue was also actively involved in hydrophobic interactions with the key residues in the active site including Phe357, Pro550 and Tyr547. Pro2 formed a hydrogen bond interaction with Tyr547 and hydrophobic interaction with Pro550, Tyr547 and Tyr666. Gln5 interacted with Arg560 via a hydrogen bond and a salt bridge. The hydrophobic associations of Trp3 in the active site of DPP IV are mediated by Phe357, Pro550, Tyr547 and Tyr629 (Figure 6C,G).

Like other peptide complexes, Tyr1′s backbone maintained a salt bridge contact and hydrogen bond with the Glu205 residue for the majority of the three runs of the DPP IV-H4 complex. In addition to this contact, Asp663 continued to have hydrogen bond and salt bridge interactions with Tyr1. This residue was also found to maintain a hydrogen bond interaction with Tyr662. This residue was also implicated in hydrophobic interactions with the active site’s important residues, including Trp201, Phe357, Pro550 and Cys551. Pro2 then maintained hydrophobic connections with Tyr547, Tyr631, Tyr662, Trp659 and Tyr666. Trp3 was associated with Tyr547 via hydrophobic interactions. Furthermore, it was observed that Thr4 maintained a salt bridge with Arg560 (Figure 6D,H).

#### 2.2.2. MD Simulations of DPP IV-Camel Hemorphin Complexes

A similar analysis was performed to evaluate the interaction of camel hemorphins in the active site of DPP IV. RMSD and RMSF values of the backbone of the three hemorphin complexes were calculated to infer stability and flexibility, indicating the inherent dynamics and fluctuations of protein while camel hemorphins were bound. For the DPP IV-camel H7 complex (Figure 4B), some deviations were observed until around 200 ns in the three runs. As expected, slight variations were observed in the RMSD values of three runs due to different sampling conditions. However, the systems were observed to be stable during the simulations. In the camel H6 complex (Figure 4D), the system was found to be equilibrated around 2.5 Å indicating a stable complex. Even though slight fluctuations in the RMSD values were observed in the camel H5 complex (Figure 4F), all runs were found to be equilibrated around 3 Å. The RMSF was calculated for all three systems and residue-level fluctuations are shown in Figure 5B,D,F. Similar to non-camel hemorphins, fluctuations were observed around residues 220–250. Ligand RMSF was also plotted for camel hemorphins and the control peptide bound to the protein (Figure S8). The plots indicated that the peptide ligands remained stably bound in the simulations.

**Figure 7:**
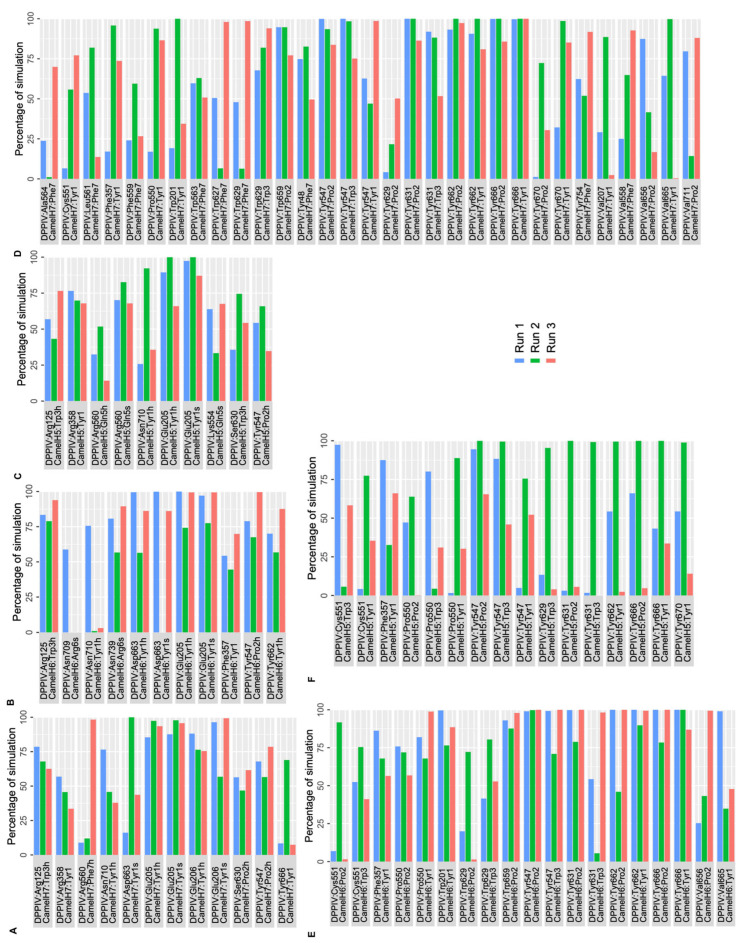
Percentage of simulation time during which intermolecular polar and hydrophobic contacts were retained between DPP IV and camel hemorphin peptides in the 500 ns systems. (**A**) Polar interaction of Camel H7 with DPP IV, (**B**) polar interaction of Camel H6 with DPP IV, (**C**) polar interaction of Camel H5 with DPP IV, (**D**) hydrophobic interaction of Camel H7 with DPP IV, (**E**) hydrophobic interaction of Camel H6 with DPP IV, (**F**) hydrophobic interaction of Camel H5 with DPP IV.

Polar and nonpolar interactions between DPP IV and camel hemorphins were analyzed. The backbone of the N terminal Tyr1 residue of camel H7 made a hydrogen bond and salt bridge interaction with Glu205 and Glu206 of the S2 pocket. Additionally, this residue was also found to form a hydrogen bond with Tyr666 and Asn710. Similar to H7, Tyr1 of camel H7 was also observed to form consistent hydrophobic interactions with S1, S1′, S2 and S2 extensive pocket residues, such as Phe357, Tyr547, Pro550, Tyr666 and Tyr662. Pro2 engaged in polar interactions with Tyr547 and Ser630 and several consistent hydrophobic interactions were observed with Tyr547, Tyr662, Tyr666, Tyr631 and Val711 of DPP IV. Tyr3 of camel hemorphin retained a hydrogen bond with Arg125 and hydrophobic contacts with Trp629, Tyr547 and Tyr631. The C terminal Phe5 interacted with Arg560 with a hydrogen bond as well formed hydrophobic interactions with Ala564, Leu561, Phe559, Trp563, Trp627 and Trp629 (Figure 7A,D).

Similar to other hemorphins, in camel H6, the N terminal Tyr1 residue interacted with Glu205 by forming hydrogen bonds and a salt bridge. In addition to this, Asp663 also interacted with the backbone of Tyr1 residue by forming hydrogen bonds and a salt bridge. Phe357, Tyr662 and Asn710 were also found to consistently interact with this residue. Apart from these interactions consistent hydrophobic connections with critical residues such as Phe357, Pro550, Tyr666 and Tyr662 also helped the peptide to stay bound in the active site. Pro2 was found to interact with Tyr547 via a hydrogen bond and interacted hydrophobically with several key residues. Arg125 maintained a hydrogen bond with Trp3 of the peptide and formed sustained hydrophobic contacts with Tyr547 and Trp629. The C terminal Arg6 interacted with Asn709 and Asn739 (Figure 7B,F).

In camel H5, the N terminal Tyr1 of H5 maintained a salt bridge and hydrogen bond interactions with Glu205. In addition to this, Arg358 and Asn710 were also found to interact with Tyr1 by retaining hydrogen bond interactions. This residue was also actively involved in hydrophobic interactions with key residues of active sites including Phe357, Pro550, Tyr547, Tyr662 and Tyr666. Pro2 interacted with Tyr547 and formed consistent hydrophobic interactions with Tyr547 and Tyr666. Tyr3 maintained hydrogen bond interaction with Arg125 and Ser630 along with hydrophobic interactions with Tyr547, Tyr629 and Tyr631. Gln5 of the peptide maintained a salt bridge with Arg560 more than 50% of simulation time in three runs. Apart from this interaction, Gln5 retained hydrogen bond contact with Lys554 and Arg560 (Figure 7C,E).

The possibility of hemorphin binding in the active site can be further determined by the MD results of the DPP IV-diprotin A system. Therefore, an MD analysis of the DPP IV-protein A complex (PDB ID:1WCY) was carried out in triplicate for 500 ns to compare the results. Stable systems were observed based on RMSD and RMSF values (Figure 4 and Figure 5H). Crucial connections with the DPP IV protein were also observed to be maintained during the simulation period. 

## 3. Discussion

Suppressing the activity of DPP IV can increase the level of active incretins, thereby decreasing blood sugar levels in diabetic patients. Therefore, it is a target for managing T2DM and efforts to find novel inhibitors are ongoing. Considering the potential side effects of chemically synthesized inhibitors, natural bioactive peptides with DPP IV inhibitory activities from various sources have been reported [32].

DPP IV inhibitory activity of peptides derived from hemoglobin and the underlying molecular mechanism of action have not been fully elucidated yet. Therefore, bioactive hemorphin peptides, including the variant from camels, were employed in this study to assess their potential inhibitory action against DPP IV. In silico methodologies involving molecular docking and MD simulation were applied to study how hemorphins could interact with DPP IV. Results suggested that, out of the 18 peptides screened, camel hemorphins H7, H6 and H5 and hemorphins H7, H6 and H5 exhibited better binding scores (GScore) and a similar binding pose in the active site of DPP IV, while LVV and VV hemorphins produced lower binding scores. Among these six peptides (H7, H6, H5, camel H7, camel H6, camel H5), hemorphin7 (H7) produced the best binding characteristics in terms of binding energy and interactions. This aligns well with the kinetic studies performed by Cohen et al.—H7 was identified to be a selective competitive inhibitor of DPP IV. However, the underlying molecular interaction of this peptide in the catalytic site of DPP IV is unclear [26]. 

Several structural and physicochemical characteristics of peptides have been proven to influence their capacity to inhibit DPP IV. Several studies have indicated that the presence of hydrophobic amino acids at the N and C terminal of peptides, a length of 2–8 amino acids, and the inclusion of a Pro residue within the peptide favor pronounced DPP IV inhibitory activity. Hemorphins are short peptides that contain hydrophobic amino acids in their N terminal, which helps their interaction in the S1 hydrophobic pocket. Additionally, the proline of hemorphins crucially engages with the hotspots of DPP IV. This finding is in line with the fact that post-proline dipeptidyl amino peptidase activity of DPP IV preferentially cleaves X-proline or X-alanine dipeptides from the N terminus of polypeptides [9]. All of the aforementioned properties of hemorphin may contribute to its DPP IV inhibitory activity. 

Ojeda-Montes and colleagues showed that compounds with aromatic rings can establish hydrophobic contacts with the S1 subsite of the DPP IV active site and are thus strong DPP IV inhibitors [13]. In addition to this, Engel et al. reported that peptides interacted with the catalytic region of DPP IV from their N terminal region [33,34]. Hemorphins (H4–H7 and camel H5–H7) have a tyrosine residue at their N-terminal. Here, these tyrosine residues were found to interact with the Glu205 and Glu206 residues of the S1 pocket via hydrogen bonds and salt bridges. These residues (Glu205 and Glu206) comprise the N-terminal recognition region of DPP IV and are thought to be the most significant anchor points for the DPP IV inhibitor recognition [3,35,36]. Furthermore, strong interaction with these critical recognition motifs leads to a significant increase in binding free energy values. Moreover, the negatively charged environment provided by these residues favors the presence of a positively charged group. In this context, the backbone amino group of Tyr was observed to form a strong noncovalent interaction with these residues (Glu205 and Glu206), and this contact significantly increases the bioactivity of DPP IV inhibitors. [36,37]. 

In addition to the above-mentioned prevalent interactions, Tyr1 of the hemorphins was observed to form interactions with Phe357 and Arg358 of the S2 extensive subsite of the DPP IV enzyme. Structure–activity studies reported that interactions with these residues can improve the activity of DPP IV inhibitors [13]. Additionally, Tyr1 also formed π–π interaction with Phe357, which can also significantly increase ligand bioactivity [38,39]. Reports suggest that interactions with the S2 extensive subsite contribute to the improved affinity of peptides [40]. The interactions with these residues were also found to be stable and consistent during molecular dynamics simulations, indicating that these contribute strongly to the binding of the peptide in the catalytic site. 

The hydroxyl group of Tyr547 formed hydrogen bonds with the Pro2 of hemorphins. The hydroxyl group of Tyr547 serves an oxyanion-stabilizing role and is thus required for the DPP IV’s catalytic activity [41,42]. Indeed, guided molecular dynamics simulations have revealed that interactions with Tyr547 are critical for retaining an inhibitor in the active site [43]. Here, Pro2 of all shortlisted hemorphins stayed strongly in contact with Tyr547 during 500 ns molecular dynamics simulations, supporting their potential DPP IV inhibitory activity. The proper orientation of the proline in the active site is achieved by their sidechain interaction with Tyr547. Pro-containing peptides are relatively resilient to gastrointestinal enzymes [44]. An analysis of reactive amino acids involved in DPP IV inhibitor peptides by Rodhi et.al revealed that proline is considered one of the most observed reactive amino acids. This is because proline forms a more stable complex with DPP IV [45]. This data may be explained by analyzing the key interactions of diprotin A (Ile-Pro-Ile), one of the most potent DPP IV inhibitory peptides [6]. Studies suggest that DPP IV has post proline dipeptidyl amino peptidase activity, preferentially cleaving X-proline or X-alanine dipeptides from the N terminus of polypeptides. Due to this, diprotin A can be hydrolyzed by DPP IV into Ile–Pro and Ile [46,47]. Additionally, it was reported that proline fits optimally in the hydrophobic active site of the protein much better than alanine. Glycine could also be accommodated in the second position but with very low k_cat_/K_M_ values [48]. Due to bulky or hydrophilic sidechains no other naturally occurring amino acid residues can occupy the second position [9].

Here, all screened hemorphins contain Tyr–Pro residues that can bind to the active core of DPP IV, limiting its catalytic activity. However, there is no Tyr-Pro at the N-terminus of LVV and VV peptides. This could explain why shorter hemorphins that lack these residues have stronger DPP IV-binding activity. Furthermore, after molecular docking, the projected orientation of the proline in the selected hemorphins resembled the orientation of the proline in the co-crystallized diprotin A in the DPP IV structure, suggesting a strong binding affinity for the hemorphins [6]. Following that, the carbonyl group of the Trp3 residue of hemorphins interacted with Arg125 of the DPP IV S2 catalytic site. Structure-activity studies have indicated that an increased affinity for DPP IV is aided by electrostatic intermolecular interaction with positively charged ligands. Here, molecular docking and simulations showed that core residues of all hemorphins, which includes the sequence Tyr-Pro-Trp-Thr (YPWT), engaged with hot spot residues of DPP IV in several favorable ways, supporting the potential to be potent DPP IV inhibitors.

Furthermore, the orientation of these hemorphins in the active site resembled that of diprotin A, supporting their strong binding affinity. This observation is strengthened by the results of the MD analysis and molecular docking studies of diprotin A. Most of the critical residue contacts required for DPP IV inhibitory activity were found to be maintained in the 500 ns simulations involving hemorphins. Our data, in agreement with the previous findings, strongly suggest that hemorphins could be a good inhibitor of DPP IV by binding strongly to its active site. This study also provides the first molecular-level insights into how this could be achieved.

## 4. Materials and Methods

### 4.1. Protein Preparation and Structure Assessment

The three-dimensional structure of DPP IV (PDB ID: 1WCY) was downloaded from the Protein Data Bank (PDB). This structure was chosen as DPP IV is complexed with the peptide diprotin A, which served as a control in this study. The protein was pre-processed using the Protein Preparation Wizard of Schrödinger Suite 2022-4 [49]. Finally, the protein structure was described using the OPLS 2005 forcefield and subsequently optimized and minimized to ensure geometric and structural stability [50].

### 4.2. Active Site Identification and Grid Generation

Before docking, a receptor grid comprising the known active site was generated. The active site residues were identified from the literature [8,51]. Additionally, residues that were observed to interact with diprotin A in the crystal structure (PDB ID: 1WCY) were also taken into consideration. For this stage, default settings and the OPLS 2005 force field were employed, with a van der Waal radius scaling factor of 1.0 to minimize penalties for near encounters and a partial charge cut-off of 0.25. Then, a cubic region appropriate for peptide docking was created around the centroid of the protein’s active site residues.

### 4.3. Peptide Docking and Binding Free Energy Calculations

Peptide docking was performed to determine the most likely binding orientation of hemorphins with DPP IV (Table S1). The extended conformations of the peptides were generated using the peptide docking panel of Schrödinger suite. The peptide docking panel of Schrödinger Glide version 2022-4 was employed to perform the docking with increased sampling and several docking runs to improve the accuracy [52]. The GlideScore (GScore) scoring method, along with free energy of binding, was used to rank the docked poses [53]. The top docked poses were shortlisted for further analysis.

### 4.4. Protein–Peptide Molecular Dynamics Simulations

The function of most proteins is dictated by their dynamics. Therefore, the dynamic motions of the docked complex were assessed to measure the strength of inter and intramolecular interactions and the complex stability. Each protein–peptide complex was subjected to 500 ns of MD simulations in triplicates [54]. All complexes were placed in an orthorhombic box of size 93 Å × 93 Å × 93 Å and solvated with single-point charge water molecules using the Desmond System Builder [55]. Subsequently, the simulation system was neutralized with the required number of counterions, and the salt concentration was set at 0.15 M NaCl. Before running the MD simulations, all systems were subjected to the steepest descent minimization and Desmond’s default eight-stage relaxation protocol. The electrostatic interactions were calculated using the Particle Mesh Ewald (PME) method with 1.0 nm short-range electrostatic and van der Waals cutoffs [56]. An NPT ensemble with the temperature at 300 K and the pressure at 1 atm was applied in all runs. The Nose–Hoover thermostat and the isotropic Martyna–Tobias–Klein barostat were used to maintain a temperature of 300 K and a pressure of 1 atm, respectively [57,58]. A time-reversible reference system propagator algorithm (RESPA) integrator was used with an inner time step of 2.0 fs and an outer time step of 6.0 fs [59]. Following the simulations, the number of protein–peptide hydrogen bonds, intermolecular interactions, root-mean-square deviation (RMSD) of the peptide and the root-mean-square fluctuation (RMSF) of both the protein and the peptide were computed. All bonds involving hydrogen atoms were constrained using the M-SHAKE algorithm implemented in Desmond [60]. Packaged and custom scripts were used to analyze the simulation data.

## 5. Conclusions

In conclusion, of the 18 hemorphin peptides docked to DPP IV, non-camel hemorphins H7, H6, H5, H4 and camel hemorphins H7, H6 and H5 had higher binding scores and similar binding orientation in the protein active region. The tetrapeptide core of all hemorphins (YPWT) docked similarly in the active site of the protein by engaging with the crucial contacts of the DPP IV protein. Compared to the control, diprotin A, hemorphins exhibited higher docking score and free energy of binding. Notably, hemorphin 7 showed higher binding affinity and sustained interactions by binding to S1 and S2 pockets of DPP IV. Furthermore, DPP IV-hemorphin complexes were found to be stable sustained throughout multiple 500 ns MD simulations by maintaining crucial interactions with the DPP IV active site residues. The presence of proline in the penultimate position of screened hemorphins facilitates stronger binding, while LVV and VV additions at the N terminal lack proline, which could account for their weak DPP IV binding. 

## Figures and Tables

**Table 1:** Interactions of the best binding pose of hemorphin peptides with DPP IV (PDB ID: 1WCY). Active site residues are in bold.

Peptide	Glide Score (GScore) Docking Score (kcal/mol)	MM-GBSA Binding Free Energy (kcal/mol)	Hydrogen Bonds	Hydrophobic Interactions	Salt Bridges	π–π Interactions
YPWTQRF (H7)	−11.44	−119.64	**Glu206**, Arg358, **Arg125, Tyr547** Asp739	Tyr48, Trp201, Val207, **Phe357**, Tyr631, Trp629, Val711, Tyr547, **Tyr666**, **Tyr662**, Trp659, Val656, Ile742, Ala743, Leu561, Leu49, Tyr752	**Glu206**, Asp739	**Phe357**
YPWTQR (H6)	−10.53	−92.92	Tyr48, Arg 358, **Glu206**, **Tyr547**, Tyr 752, His748, Ala743	Val207, **Phe357, Tyr547**, Tyr631, Trp629, Trp627, **Tyr662**, Trp659, Val656, **Tyr666**, Val711, Tyr752, Tyr48, Ala743	**Glu205**	**Phe357**
YPWTQ (H5)	−10.76	−99.12	**Glu206**, Arg358, **Tyr547, His740**, **Arg125**, Gly741	Val207, Trp201, **Tyr547**, Ile742, Ala743, Tyr752, Tyr48, **Tyr662**, Trp629, Trp659, Tyr631,Trp659, Val711, Val656, **Tyr666**, **Phe357**	**Glu206**	**Phe357,** Trp629
YPWT (H4)	−7.6	−84.98	Arg358, **Glu206, Glu205, Tyr547, Arg125, His740**	Trp201, **Tyr547**, Val207, Val656, **Phe357**, Trp659, **Tyr666, Tyr662,** Val711, Tyr631, Trp629	**Glu205**	**Phe357**
YPWTRRF (Camel H7)	−10.54	−113.28	Arg358, **Glu206, Tyr547, His740, Arg125**	Val207, Trp201, **Tyr547**, Trp563, Tyr752, Tyr48, Leu55, Leu57, Val656, Trp659, Tyr631, Trp659, Val711, **Tyr666, Phe357, Tyr662**	**Glu206**	**Phe357**
YPWTRR (Camel H6)	−10.07	−85.99	Arg358, **Glu206, Tyr547, His740, Arg125**	Val207, Trp201, **Tyr547**, Tyr120, Tyr128, **Phe357,** Ala743, Val656, Trp659, **Tyr662**	**Glu206**	**Phe357**
YPWTR (Camel H5)	−9.42	−102.88	**Arg125, Tyr547**	Trp201, **Tyr547**, Ile742, Ala743, Tyr752, Phe357, **Tyr666**, Val656, Tyr631, Trp659, Val711, Trp629, **Tyr662**	**Glu206**	**Phe357**
Diprotin A (Positive control)	−8.05	−83.00	**Arg125, Tyr547,** Tyr631	**Phe357,** Tyr662, **Tyr666,** Trp659, Val711, **Tyr547,** Tyr631, Trp629	**Glu 205, Glu 206**	

## Data Availability

Data is contained within the article and Supplementary Materials.

## Supplementary Materials

The following supporting information can be downloaded at: https://www.mdpi.com/article/10.3390/ijms25053059/s1.
