# Supplementary material for: Computational Modeling of the Interactions between DPP IV and Hemorphins
Source: Int J Mol Sci. 2024 Mar 6;25(5):3059. doi: 10.3390/ijms25053059 (PMC10932442; doi:10.3390/ijms25053059)
Supplement: Supplementary file 1 [file ijms-25-03059-s001.zip › ijms-2844285-supplementary.pdf]

Computational modeling of the interactions between DPP IV and hemorphins

Priya Antony[1], Bincy Baby[1], Amie Jobe[1] and Ranjit Vijayan[1,2,3,*]

[1] Department of Biology, College of Science, United Arab Emirates University, PO Box 15551, Al Ain, United Arab Emirates
[2] The Big Data Analytics Center, United Arab Emirates University, PO Box 15551, Al Ain, United Arab Emirates.
[3] Zayed Center for Health Sciences, United Arab Emirates University, PO Box 15551, Al Ain, United Arab Emirates.
* Corresponding authors: ranjit.v@uaeu.ac.ae (R.V.)

Supplementary Materials

Table S1: List of peptides used in this study

#	**Peptides**	**Amino acids**
1	YPWTQRF (H7)	Tyr-Pro-Trp-Thr-Gln-Arg-Phe
2	YPWTQR (H6)	Tyr-Pro-Trp-Thr-Gln-Arg
3	YPWTQ (H5)	Tyr-Pro-Trp-Thr-Gln
4	YPWT (H4)	Tyr-Pro-Trp-Thr
5	VVYPWTQR (VVH6)	Val-Val-Tyr-Pro-Trp-Thr-Gln-Arg
6	VVYPWTQRF (VVH7)	Val-Val-Tyr-Pro-Trp-Thr-Gln-Arg-Phe
7	LVVYPWT (LVV H4)	Leu-Val-Val-Tyr-Pro-Trp-Thr
8	LVVYPWTQ (LVV H5)	Leu-Val-Val-Tyr-Pro-Trp-Thr-Gln
9	LVVYPWTQR (LVV H6)	Leu-Val-Val-Tyr-Pro-Trp-Thr-Gln-Arg
10	LVVYPWTQRF (LVV H7)	Leu-Val-Val-Tyr-Pro-Trp-Thr-Gln-Arg-Phe
11	YPWTRRF (Camel H7)	Tyr-Pro-Trp-Thr-Arg-Arg-Phe
12	YPWTRR (Camel H6)	Tyr-Pro-Trp-Thr-Arg-Arg
13	YPWTR (Camel H5)	Tyr-Pro-Trp-Thr-Arg
14	VVYPWTRR (Camel VVH6)	Val-Val-Tyr-Pro-Trp-Thr-Arg-Arg
15	VVYPWTRRF (Camel VVH7)	Val-Val-Tyr-Pro-Trp-Thr-Arg-Arg-Phe
16	LVVYPWTR (Camel LVV H5)	Leu-Val-Val-Tyr-Pro-Trp-Thr-Arg
17	LVVYPWTRR (Camel LVV H6)	Leu-Val-Val-Tyr-Pro-Trp-Thr-Arg-Arg
18	LVVYPWTRRF (Camel LVV H7)	Leu-Val-Val-Tyr-Pro-Trp-Thr-Arg-Arg-Phe

Table S2: GlideScore (GScore) and binding energy values of VV and LVV hemorphins

Peptides	GScore binding score (kcal/mol)	MM-GBSA binding free energy (kcal/mol)
VVYPWTQR (VVH6)	-7.82	-92.89
VVYPWTQRF (VVH7)	-8.49	-91.34
LVVYPWT (LVV H4)	-8.31	-87.75
LVVYPWTQ (LVV H5)	-9.94	-91.11
LVVYPWTQR (LVV H6)	-9.24	-94.30
LVVYPWTQRF (LVV H7)	-10.60	-93.14
VVYPWTRR (Camel VVH6)	-9.93	-95.66
VVYPWTRRF (Camel VVH7)	-9.76	-92.56
LVVYPWTR (Camel LVV H5)	-9.87	-98.47
LVVYPWTRR (Camel LVV H6)	-9.62	-95.11
LVVYPWTRRF (Camel LVV H7)	-10.39	-92.35

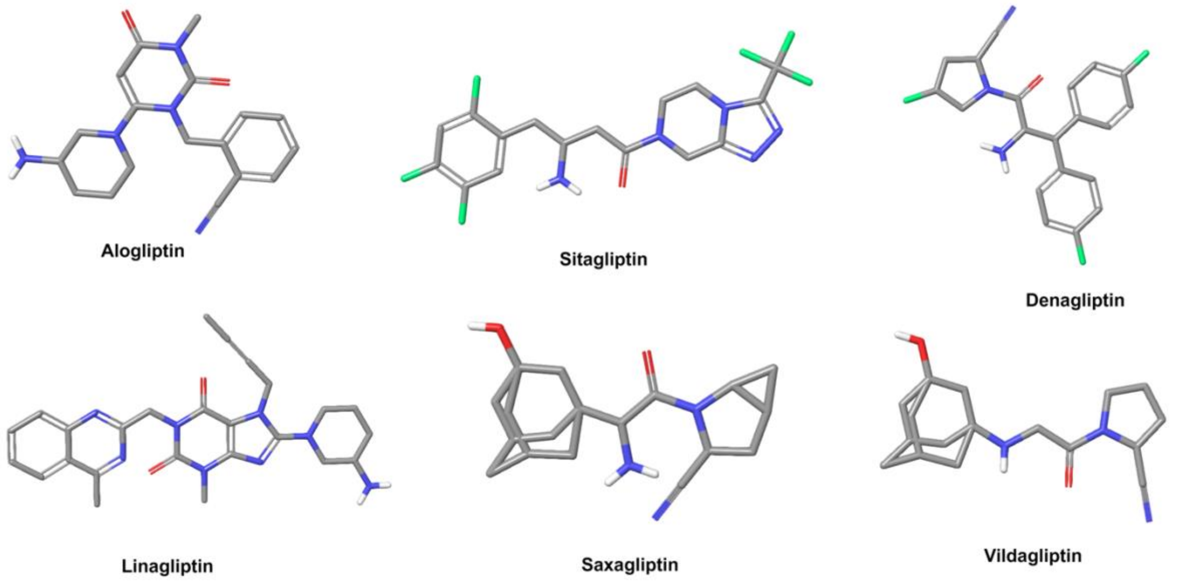

Figure S1: Chemical structures of DPP IV inhibitors.

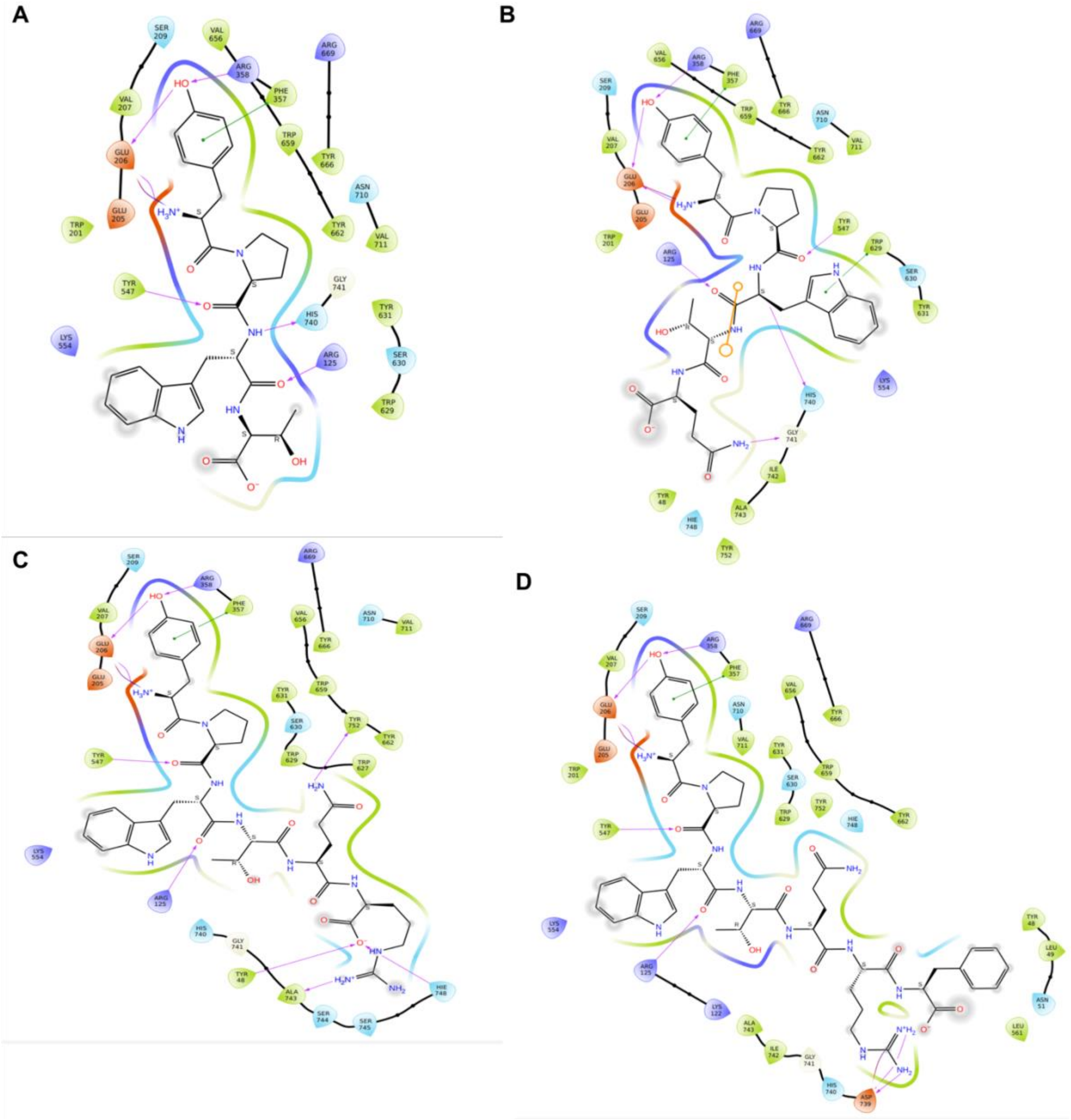

Figure S2: Ligand interaction diagram of hemorphins (A) H4 (B) H5 (C) H6 (D) H7.

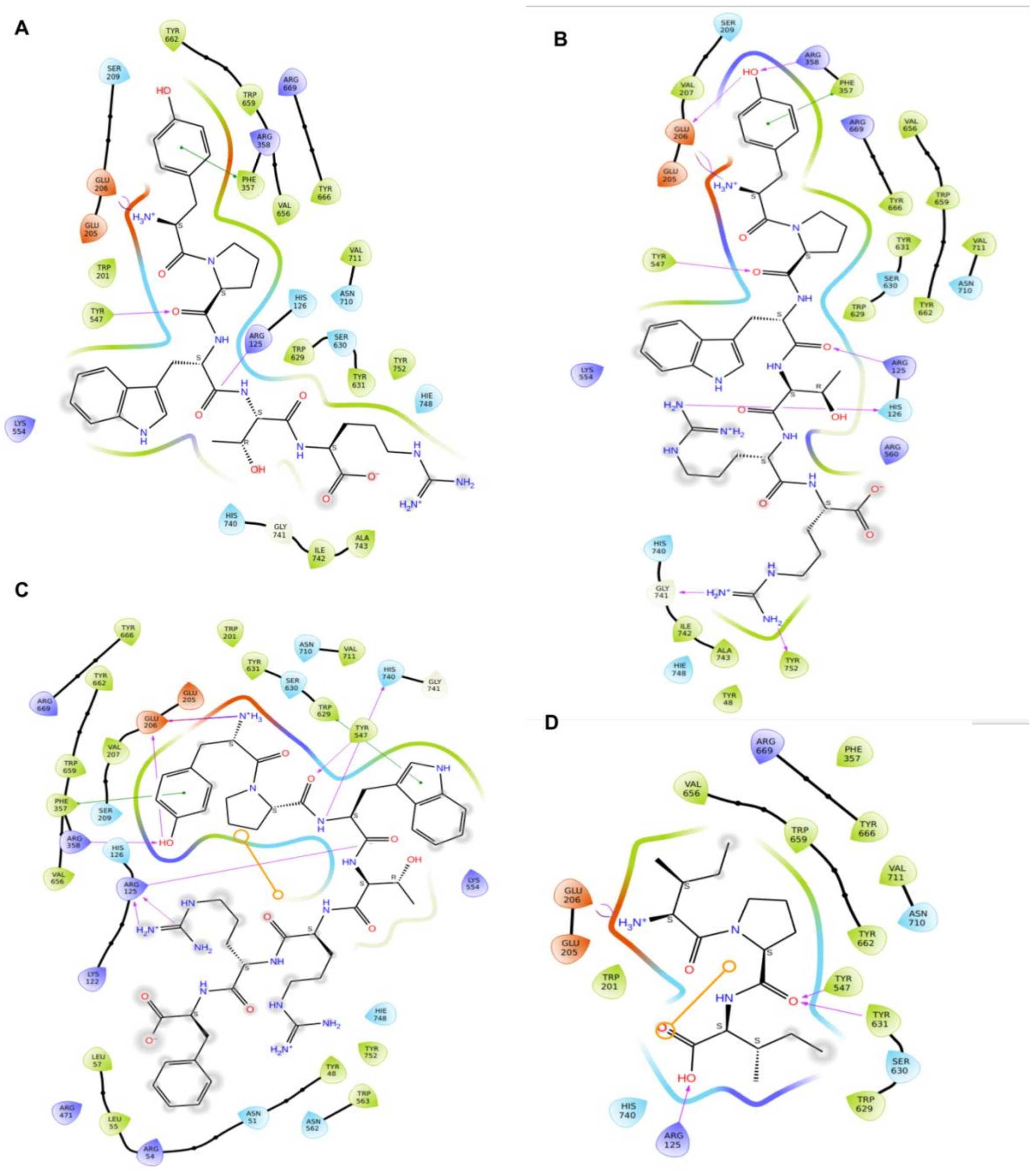

Figure S3: Ligand interaction diagram of camel hemorphins and control (A) Camel H5, (B) Camel H6, (C) Camel H7, (D) Diprotin A

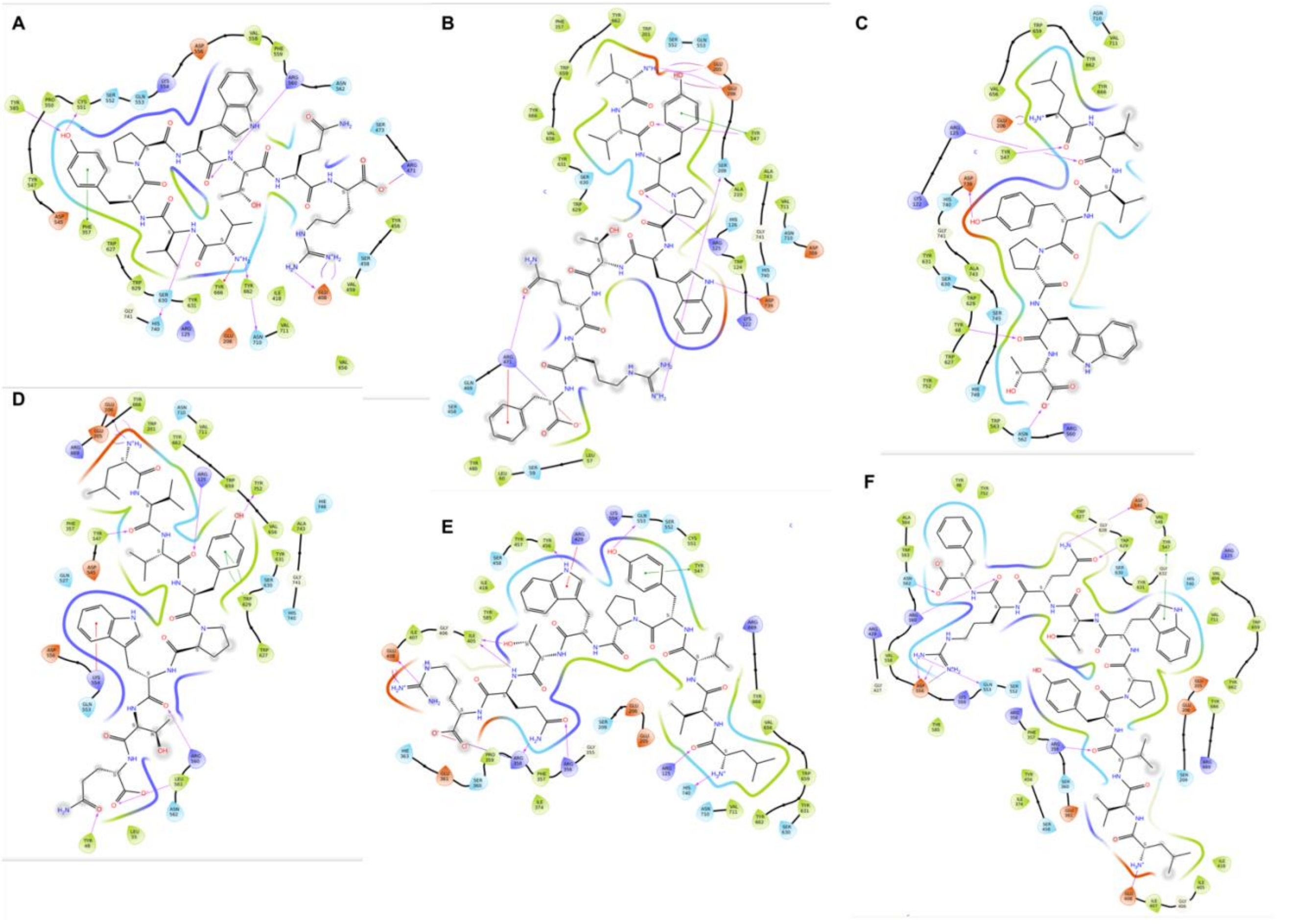

Figure S4: Ligand interaction diagram of hemorphins (A) VVH6 (B) VVH7 (C) LVVH4 (D) LVVH5 (E) LVVH6 (F) LVVH7

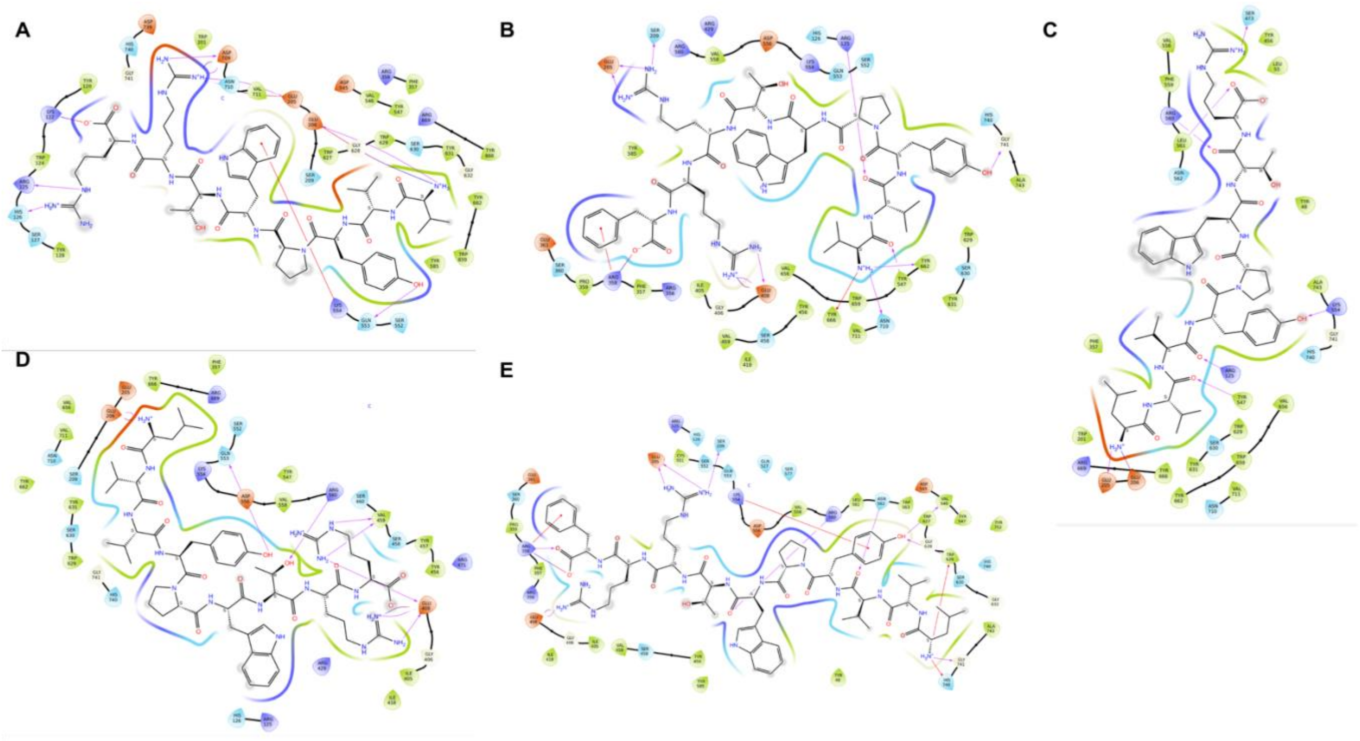

Figure S5: Ligand interaction diagram of camel hemorphins (A) Camel VVH6 (B) Camel VVH7 (C) Camel LVVH5 (D) Camel LVVH6 (E) Camel LVVH7

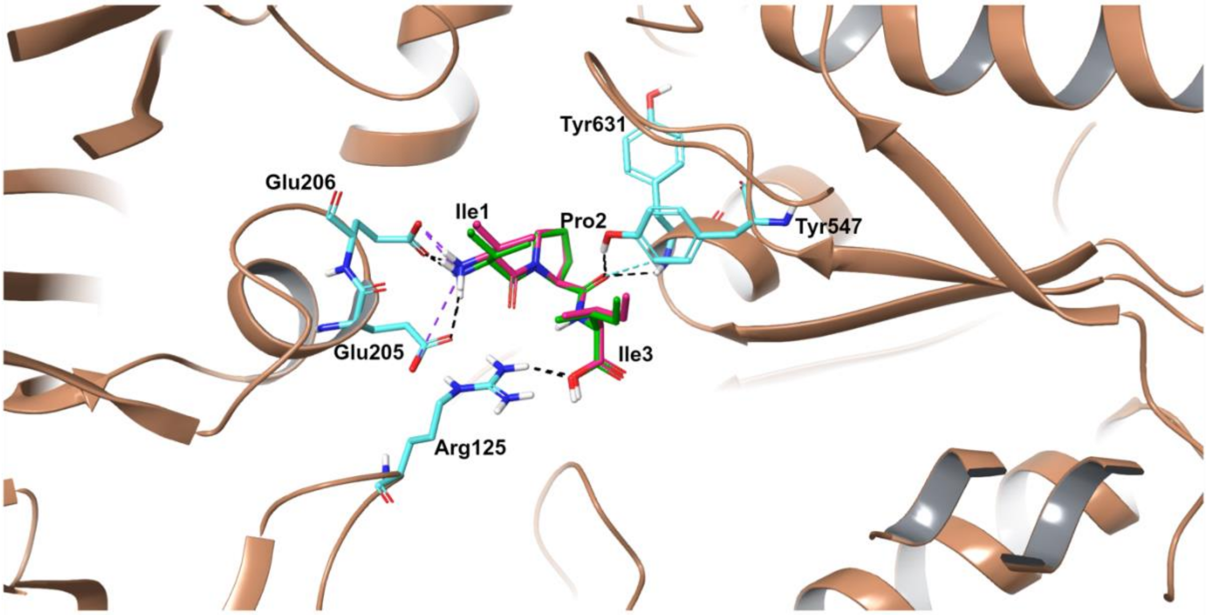

Figure S6: Superimposed structure of positive control Diprotin A in docked conformation (pink colour) and experimental position (green colour).

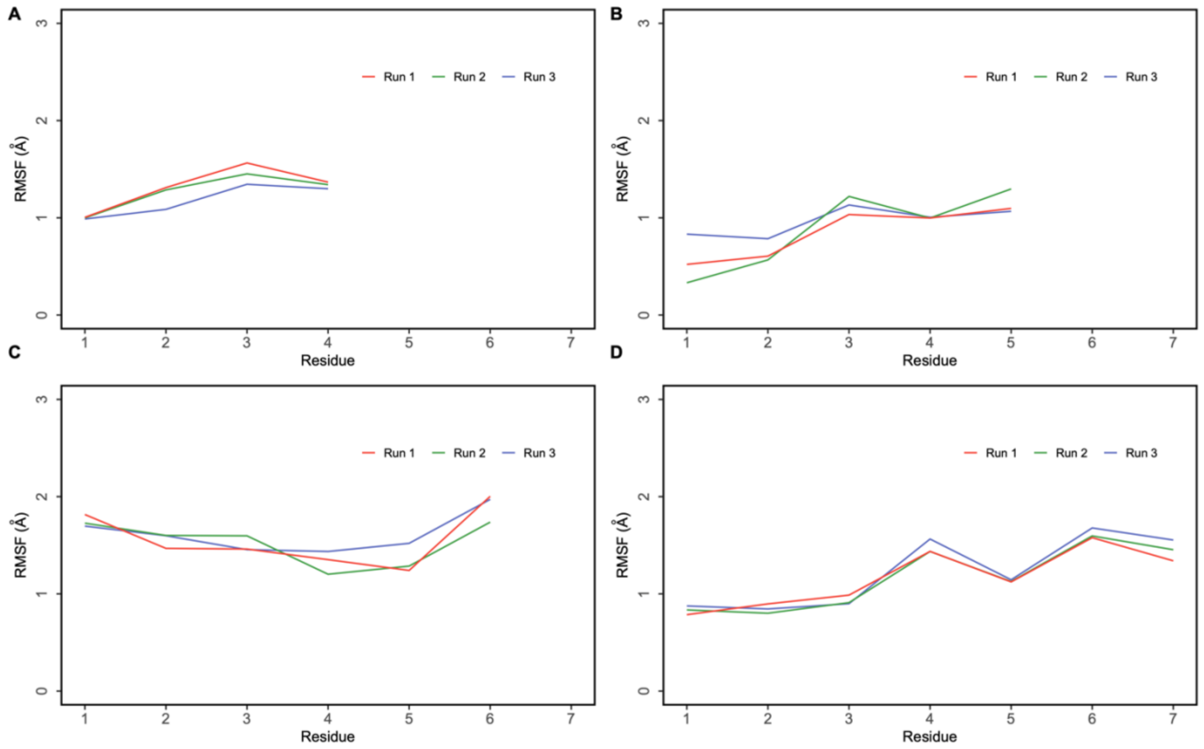

Figure S7: Root Mean Square Fluctuation (RMSF) of bound peptide Cα atoms after aligning the protein structure in each frame of the simulation. Non-camel hemorphins (A) H4, (B) H5, (C) H6, (D) H7.

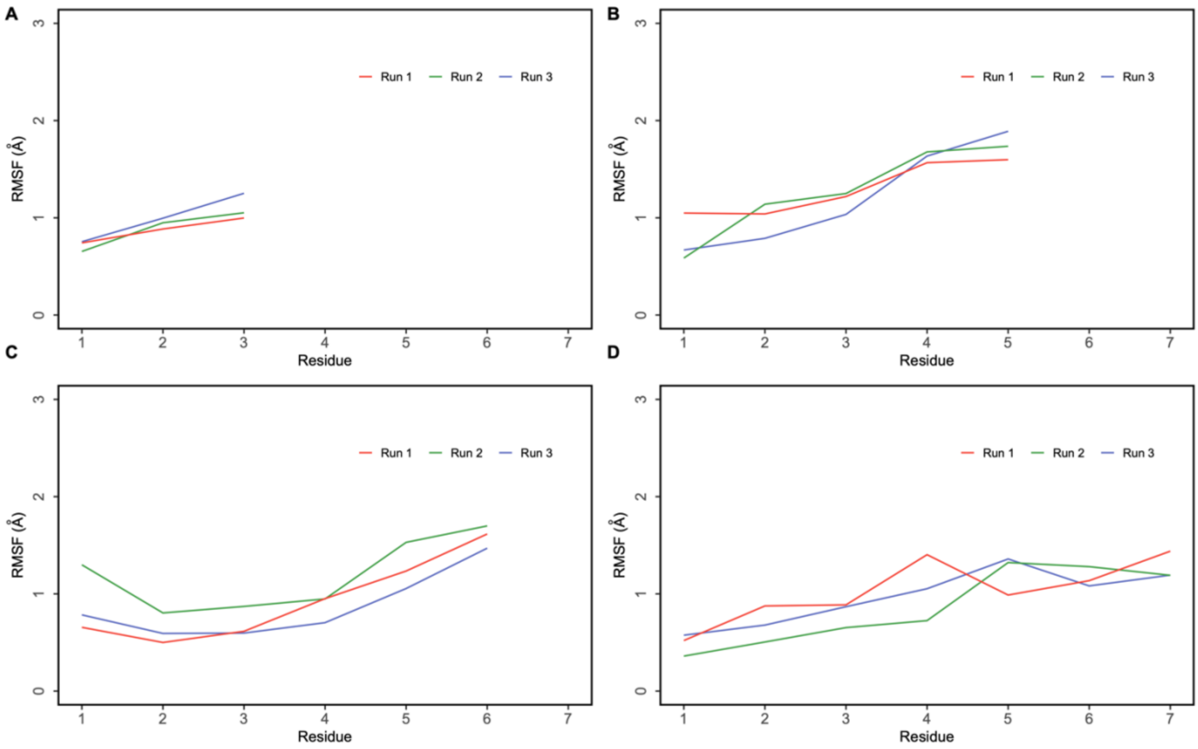

Figure S8: Root Mean Square Fluctuation (RMSF) of bound peptide Cα atoms after aligning the protein structure in each frame of the simulation. (A) Diprotin A, (B) Camel H5, (C) Camel H6, (D) Camel H7.
